# Managing Hypoglycaemia in Patients With Insulinoma—A Tertiary Centre Experience and Review of the Literature

**DOI:** 10.1111/cen.15188

**Published:** 2024-12-30

**Authors:** Sophie Howarth, Tak‐Wai Ho, James Wimbury, Ruth Casey

**Affiliations:** ^1^ University of Cambridge Cambridge UK; ^2^ Department of Diabetes and Endocrinology Cambridge Cancer Centre and Cambridge University Hospitals NHS Foundation Trust Cambridge UK; ^3^ Department of Nutrition and Dietetics Cambridge University Hospitals NHS Foundation Trust Cambridge UK; ^4^ Department of Medical Genetics University of Cambridge and NIHR Cambridge Biomedical Research Centre Cambridge UK

**Keywords:** 177Lu‐DOTATATE, diazoxide, everolimus, hypoglycaemia, insulinoma, neuroendocrine tumour, peptide receptor radionuclide therapy, somatostatin analogue

## Abstract

The management of hypoglycaemia is pivotal in the care of patients with insulinoma. Blood glucose monitoring and regulation needs careful attention pre‐ and peri‐operatively for patients undergoing surgical resection and as part of the long‐term management for patients with inoperable or metastatic disease. Hypoglycaemia symptoms are frequently pervasive and disabling, with many patients showing impaired hypoglycaemia awareness that can lead to life‐threatening severe hypoglycaemia. Herein, we review the literature and describe our tertiary centre experience in the mutli‐disciplinary management of hypoglycaemia for patients with proven insulinomas. We propose a stepwise algorithm for the management of hypoglycaemia, stratified by localised versus metastatic disease. We discuss our strategy for the nutritional management of hypoglycaemia, reviewing the evidence for the use of cornstarch products and artificial nutrition. We discuss pharmacological management including diazoxide, somatostatin receptor antagonists (SSAs), everolimus and glucocorticoids, in addition to other therapeutic interventions such as peptide receptor radionuclide therapy (PRRT) and endoscopic ablation.

## 1 Introduction

Hypoglycaemia associated with insulin‐secreting pancreatic neuroendocrine tumours was first reported in the early 20th century [1, 2]. The presence of neuroglycopenic symptoms in association with fasting hypoglycaemia (plasma glucose concentration < 3 mmol/L or < 55 mg/dL) and resolution of these symptoms after ingestion of glucose is termed ‘Whipple's triad’. Concomitant hypoglycaemia, elevated insulin, pro‐insulin, and C‐peptide is indicative of endogenous hyperinsulinism, warranting radiological investigations to identify the tumour source. Computed tomography (CT) pancreas, endoscopic ultrasound ± fine needle aspiration, magnetic resonance imaging (MRI) and functional imaging methods like Gallium‐68‐DOTATATE PET CT, which binds to somatostatin receptor subtype 2 (SSTR2), are employed in localising insulinomas. Insulinomas are rare tumours with an incidence of 1−4/million person years [3], and other causes of adult‐onset hypoglycaemia should be excluded. This includes insulin‐mediated causes of hypoglycaemia such as exogenous sulphonylurea or insulin administration, insulin or insulin receptor antibodies, renal failure and post‐gastric bypass syndrome, and non‐insulin mediated causes like hepatic failure, malnutrition, adrenal insufficiency, and insulin‐like growth factor secreting tumours.

Hypoglycaemia symptoms in patients with insulinoma are frequently pervasive and disabling. The autonomic symptoms of hunger, sweating, weakness, dizziness and confusion are commonly present at diagnosis, but their non‐specific nature means diagnosis is often delayed [4]. The development of the hypoglycaemic syndrome several years after a diagnosis of non‐functioning pancreatic neuroendocrine tumour has been reported and is associated with a worse prognosis [5, 6]. Hypoglycaemic awareness can decline over time, putting patients at risk of severe life‐threatening hypoglycaemic events [7].

Most insulinomas are benign, single tumours and for many patients surgical resection is curative [4]. Pre‐ and peri‐operative glycaemic control remains a crucial part of management. For the 5−10% of patients with malignant disease [3, 8], those with unresectable or ectopic disease and those with co‐morbidities precluding surgery, long term management of hypoglycaemia is the focus of treatment. Achieving normoglycaemia requires a multi‐modal therapeutic approach and can be challenging, with one single‐centre study of treated endogenous hypoglycaemia reporting ongoing asymptomatic hyper or hypoglycaemic deviations in 56% of patients [9].

Herein, we report our multi‐disciplinary tertiary centre experience in managing these patients, review the literature for each treatment modality and propose an algorithm for the stepwise management of hypoglycaemia in patients with hyperinsulinemia caused by an insulin secreting neuroendocrine tumour.

## 2 Methods

We performed a search of the literature available on PubMed up until October 2024. We excluded non‐human studies and non‐English language articles. Articles identified through bibliographies were also included.

### 2.1 Case Examples

#### 2.1.1 Patient 1

A lady in her 70 s was admitted to her local hospital following a loss of consciousness associated with severe hypoglycaemia (capillary blood glucose 0.8 mmol/L). CT pancreas failed to identify a pancreatic tumour, but endoscopic ultrasound revealed a culprit 8 mm lesion in the tail of the pancreas with biopsy suggestive of a grade 1‐well‐differentiated neuroendocrine tumour. She was started on diazoxide as an inpatient, but this was stopped after a few days due to fluid retention and 3.5 kg weight gain. Octreotide 50 mg/8 h was then trialled, but she experienced steatorrhoea and nausea with no improvement in her glycaemic control. Subsequent specialist neuroendocrine dietitian input and a trial of cornstarch 50 g with yoghurt at bedtime resulted in a significant reduction in episodes of severe hypoglycaemia. She underwent successful distal pancreatectomy with normalisation of glycaemic control post‐operatively.

#### 2.1.2 Patient 2

**Figure 1:**
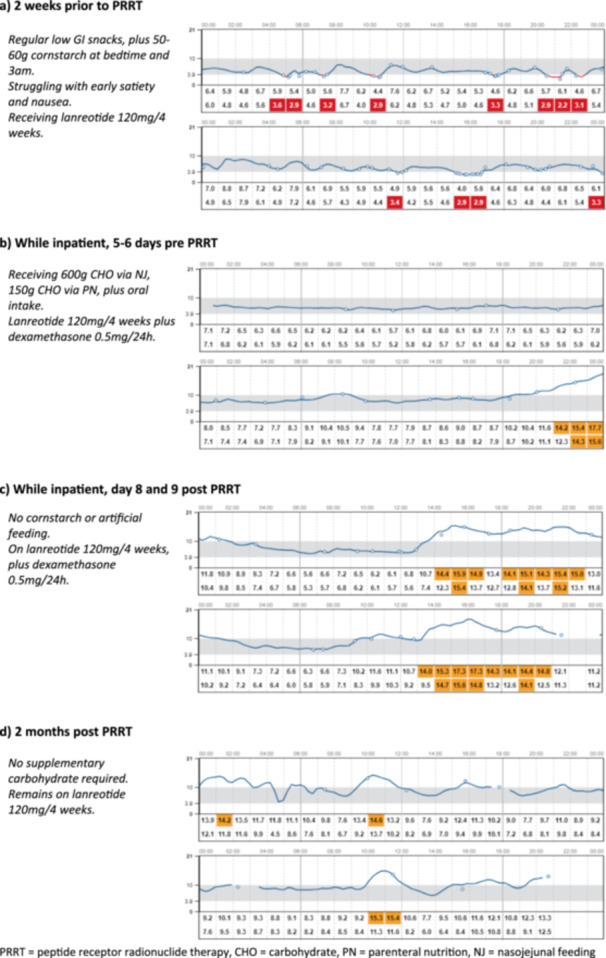
Continuous glucose monitoring data pre and post peptide receptor radionuclide therapy (PRRT).

A man in his 60 s presenting with abdominal pain, loose stools, 9 kg weight gain and recurrent hypoglycaemia was found to have an inoperable ill‐defined mass in the body of the pancreas encasing splenic vessels, with liver lesions and mesenteric nodal disease. Gallium‐68‐DOTATATE imaging and endoscopic ultrasound (EUS) biopsy confirmed the diagnosis of metastatic insulinoma. CGM monitoring showed impaired hypoglycaemia awareness. He was referred to our specialist neuroendocrine dietitians who started 50−60 g cornstarch (mixed with yoghurt) at bedtime and 3am, alongside pancreatic enzyme replacement therapy. Diazoxide was contraindicated due to concurrent ascites, hence he was started on Lanreotide Autogel 120 mg 4 weekly. Despite this, he continued to have multiple episodes of hypoglycaemia and early satiety prevented further increase in his oral intake. He was admitted to hospital and ultimately required simultaneous naso‐jejunal feeding, 10% dextrose intravenous infusion at 100 ml/h and parenteral nutrition to prevent hypoglycaemia. He underwent his first cycle of peptide receptor radionuclide therapy (PRRT) during his inpatient stay. Over the following 7 days, all supplementary NJ feeding, PN and IV dextrose were weaned, and he was discharged on Day 8 with Lanreotide 120 mg/4 weekly, pancreatic enzyme replacement therapy and dexamethasone 0.5 mg daily. His CGM data pre and post‐PRRT is summarised in Figure 1.

### 2.2 Monitoring

#### 2.2.1 Continuous Glucose Monitoring

Patients with insulinoma frequently have impaired hypoglycaemia awareness and evaluating treatment effect by symptoms alone may miss asymptomatic hypoglycaemia [7, 9]. Continuous glucose monitoring devices offer non‐invasive real‐time blood sugar monitoring and alarms which can be set to alert patients to low or falling blood glucose before severe hypoglycaemia occurs. They have been shown to reduce the time spent in hypoglycaemia and severe hypoglycaemic events in children and adults with type 1 diabetes [10, 11, 12] and they have been reported to demonstrate cure following surgical intervention in patients with insulinoma [7, 13].

### 2.3 Management

#### 2.3.1 Nutrition

Carbohydrates affect blood glucose levels more than any other macronutrient and as such should be the primary consideration alongside the frequency of meals and snacks. The impact that carbohydrates have on glucose levels is principally influenced by the quantity consumed and the rate at which it is digested and metabolised. The glycaemic index (GI) describes the theoretical rate at which carbohydrates increase blood glucose levels, with higher GI indicating a faster rise in glucose levels, and this can be influenced by the type and structure of the carbohydrate, processing and cooking method, as well as the presence of other nutrients such as protein, fat and fibre [14, 15].

A thorough dietary history, such as a 24‐h dietary recall, should be completed to assess the current dietary adequacy. Due to the importance of carbohydrates in maintaining euglycaemia, thorough education should be provided to the patient on carbohydrate types, GI, how to read food labels, and how to quantify carbohydrates where appropriate.

First line advice for patients who can take nutrition orally is to follow a lower GI diet with regular meals and snacks. In our centre, a starting point of 1 g of carbohydrate per kg body weight every 4−6 h is used to estimate the approximate amount of carbohydrate required to prevent hypoglycaemia; this figure is taken from UK dietetic guidance for managing glycogen storage disease [16]. For individuals with a body mass index (BMI) of 30 kg/m^2^ or higher, we use the ideal body weight (IBW) using a BMI of 25.0 kg/m^2^ in calculations. If hypoglycaemia occurs overnight, it may be necessary for an individual to take a carbohydrate containing snack during the night. If the starting dose of carbohydrate taken every 4−6 h is insufficient to prevent hypoglycaemia, then adjustments should be made dependent on the pattern of hypoglycaemia. This can include increasing the amount of carbohydrate at specific times of day and decreasing the time between carbohydrate containing snacks/meals, though other techniques such as increasing protein intake can also be beneficial as it is metabolised slowly into glucose and may aid in reducing secondary hypoglycaemia from reactive insulin stimulation [17].

The introduction of uncooked cornstarch (UCCS), or modified cornstarch products such as Glycosade, can be considered if hypoglycaemia persists despite first‐line management. Supporting evidence for their benefit in sustaining euglycaemia is drawn from studies in glycogen storage disease type 1 (GSD1), where UCCS prevented hypoglycaemia for between 4 and 6 h, and Glycosade for between 6.5 and 7.8 h [18, 19]. While no clinical trials have explicitly examined how long UCCS or modified cornstarch products can maintain euglycaemia in individuals with insulinomas, Li et al. demonstrated that UCCS taken every 6 h during the daytime can increase fasting glucose levels, as well as overnight when taken before bed or every 4 h [20]. Practical considerations include the palatability of the product, which can be mixed with other foods or drinks depending on individual preference, as well as tolerance as large amounts of undigestible carbohydrate can cause gastrointestinal side effects such as bloating, abdominal pain and diarrhoea [21]. In this scenario anti‐diarrhoeal agents may be considered to alleviate symptoms. While there is limited direct evidence on the impact of combining UCCS or modified cornstarch products with different foods, in our experience mixing UCCS or modified cornstarch products with macronutrient rich foods such as milk or yoghurt rather than water may increase the likelihood of maintaining euglycaemia.

The combination of increased calorie intake, frequent hypoglycaemia treatments and the anabolic nature of increased endogenous insulin production usually leads to weight gain in these individuals. The priority of care is avoiding life threatening hypoglycaemia, hence reducing carbohydrate intake should be avoided. Reducing fat intake to minimise weight gain may be the safest approach, as this has a limited impact on glucose levels compared to carbohydrate and is more calorie dense. Dietary changes should be made with dietitian supervision.

Patients should be educated on hypoglycaemia treatment at the earliest opportunity. The limited evidence base and lack of consensus regarding hypoglycaemia treatment for insulinoma patients means treatment guidelines for patients with diabetes are widely adapted and used in practice, with the threshold for treatment at ≤ 4 mmol/L. The treatment dose of rapid acting carbohydrates used may be tailored to the individual based on historical response to hypoglycaemia using capillary glucose testing. Whereas intramuscular glucagon is indicated for the treatment of severe hypoglycaemia in diabetes, usage in individuals with insulinomas is contraindicated for most patients due to the risk of reactive hypoglycaemia following initial hyperglycaemia [22]. If intramuscular glucagon is felt to be appropriate, education on how and when to administer the dose should be provided to the patient and their next of kin.

Artificial nutrition (AN) such as enteral (EN) or parenteral (PN) nutrition may be required when oral nutritional support options have been exhausted [23]. Indications for AN include early satiety and lack of appetite, nausea and/or vomiting, malabsorption and above all intractable hypoglycaemia [17]. Nocturnal AN may be suitable for patients who are able to prevent hypoglycaemia in the daytime but find it a challenge to eat enough at bedtime/overnight [23, 24, 25]. The practicalities of delivering AN at home should be considered as soon as the need is identified, but in reality, patients may require a period of hospitalisation with IV dextrose administration while AN is established to avoid life threatening hypoglycaemia.

When developing an enteral feeding regimen for insulinoma patients, consideration should be given to the route, composition and timing of feeding. Though there is a lack of supporting evidence, it is hypothesised that the most beneficial type of enteral feed should be high in carbohydrate, fibre and protein and low in calories and volume. We assess diet quality to assist in the decision to use a nutritionally complete feed or a modular individual carbohydrate feed. Modular carbohydrate feeds may be the feed of choice for patients who are meeting their non carbohydrate nutritional requirements and the goal is to minimise excessive calories. The quantity of carbohydrate should depend on the severity and frequency of hypoglycaemia; if intravenous dextrose has been used, it is useful to calculate how much carbohydrate is given per hour and match this quantity. The use of a diabetes‐specific or fibre containing feed may be useful for its low GI properties [26]. This is especially important when jejunal feeding is used, as the risk of reactive hypoglycaemia is greater due to a more pronounced insulin response during jejunal feeding [27, 28]. Percutaneous gastrostomy/jejunostomy (PEG/PEJ) feeding may be appropriate when long term EN is expected [29]. Ongoing dietetic review to adjust carbohydrate quantities is essential and can be aided by use of glucose sensor technology. There should always be a clear contingency plan in the event of tube displacements and feed interruptions.

Though there is limited evidence for its use in patients with insulinoma, in cases where malabsorption or recurrent vomiting prevents enteral feeding, PN may be required. If PN is deemed necessary, it is important to liaise closely with the local PN team to calculate carbohydrate quantities and to choose appropriate PN formulations. Our practice is to use individually compounded mixtures to enable tailoring of carbohydrate quantities, while concurrently making a fat free PN solution. The PN team can help to guide the need for additional nitrogen, vitamin and minerals as well as provide ongoing monitoring of electrolyte, fluid and nutritional status [29]. However, the adjustment of carbohydrate within the PN should be calculated by the treating team, based on the patient's glycaemic control. Proactive glycaemic review and regular communication with the PN team is crucial to allow time for changes in the PN formulations to be made up. We have experience of PN causing reactive hypoglycaemia at the start and end of the feed. This has been overcome by using a gradual taper up and taper down of PN over a duration of 2 h.

Setting up home PN requires training of the patient, family members or carers and contingency planning [29]. Close MDT working with the home PN team is paramount in achieving safe and effective maintenance of euglycaemia. Delays in changing PN formulation can be overcome by creating bags with higher than required carbohydrate quantities so that an increase in infusion rate can be instigated without the need to wait for a change in the PN formulation.

#### 2.3.2 Pharmacotherapy

The key properties of the different pharmacological agents are summarised in Table 1.

**Table 1:** Pharmacological therapies for insulinoma.

	Mechanism of action	Dose	Time to achieve euglycaemia	Time remaining in euglycaemia	Reported adverse effects
Diazoxide	Direct K_ATP_ channel potentiation in pancreatic β cells	3−8 mg/Kg PO titrated to therapeutic effect. Doses up to 20 mg/Kg reported [25, 30]	2−3 weeks [25]	Long term treatment up to 27 years has been reported [31]	Fluid retention (> 50%), thrombocytopenia, hirsutism, rash
Somatostatin analogues	Agonists of G‐protein coupled receptors SSTR1‐5			Long term euglycaemia from 15−67 months reported [32, 33]	Nausea, abdominal discomfort, diarrhoea, steatorrhoea, flatulence, cholelithiasis, paradoxical hypoglycaemia with dose titration
Octreotide	Highest affinity for SSTR2	Short acting: 100−600 mcg/24 h SC in 2−4 divided doses Long acting: 20−30 mg every 4 weeks IM	Short acting: 6−8 h [34] Continue short acting form for 2 weeks after first dose of LAR [25]		
Lanreotide	Highest affinity for SSTR2	Long acting: 60−120 mg SC every 4 weeks			
Pasireotide	Higher affinity for SSTR5, also SSTR2, SSTR3, SSTR1 [35]	Short acting: 0.9 mg/12 h SC [36] Long acting: 60 mg SC every 4 weeks [37]			
Everolimus	mTOR (mammalian target of rapamycin) inhibitor	5−10 mg PO once daily	3−14 days [38, 39]	6−12 months [39, 40]	Rash (49%), diarrhoea (35%), stomatitis, fatigue, infections [41]
Glucocorticoids	Inhibition of insulin release and increased peripheral insulin resistance	Prednisolone 2.5 mg PO daily Dexamethasone 0.5−4 mg PO daily	Uncertain	Uncertain	Hyperglycaemia, reduced bone mineral density, thinning of skin, GI upset including bleeding, secondary adrenal insufficiency
Peptide receptor radionuclide therapy (PRRT)‐177Lu‐DOTATATE (Lutathera)	Peptide somatostatin receptor agonist DOTA‐TATE and 177‐Lutetium	4 doses of 7.4 GBq, given as an IV infusion at 8 week intervals—growing evidence for efficacy of further maintenance doses	Most patients (65% of all patients, 81% of responders) within 1−2 weeks of cycle 1 [42]	1−2 years [42, 43, 44, 45]	Nausea/vomiting post infusion, transient myelotoxicity, transient worsening of hypoglycaemia

##### 2.3.2.1 Diazoxide

Diazoxide is a non‐diuretic benzothiadiazine derivative first introduced in clinical practice for the management of hypertension and utilised in the treatment of insulinomas since the late 1970s. Diazoxide is typically initiated at a starting dose of 3−8 mg/Kg/day titrated to therapeutic effect, though doses of up to 20 mg/kg/day for refractory hypoglycaemia have been reported [25, 30]. Diazoxide acts directly on pancreatic beta cells, potentiating the opening of K_ATP_ channels and suppressing insulin secretion [46]. Cohort studies report an improvement in glycaemic control for around half of all patients, with 59% of patients reportedly ‘symptom free’ with long term diazoxide treatment in Gill et al. [47] and 9 out of 20 patients treated with diazoxide able to fast for 10−12 h without the need for IV glucose infusion in Niitsu et al. [30]. Diazoxide may be more effective in those with smaller tumour size, though this association was not statistically significant after the exclusion of patients with metastatic disease in the study by Niitsu et al. [30]. Long term diazoxide treatment of up to 27 years has been reported [31]. However, the direct vasodilatory effect of diazoxide on vascular smooth muscle can cause progressive fluid retention, alongside other side effects such as hirsutism, rash and thrombocytopenia [30, 48, 49]. Over half of the patients in the study by Niitsu et al. discontinued treatment due to unacceptable side effects and 55% of patients in the study by Gill et al. were prescribed concurrent diuretics. Thiazide diuretics are the diuretic of choice due to their hyperglycaemic effect [50]. Both the therapeutic effect of diazoxide and the side effects of fluid retention and thrombocytopenia are typically observed in the first 2−3 weeks [30, 48, 51].

##### 2.3.2.2 Somatostatin Analogues

The somatostatin receptors, of which there are 5 subtypes (SSTR1‐5), are G‐protein coupled receptors that offer a diagnostic and therapeutic target for patients with insulinoma. The majority of insulinomas show immunohistochemical staining for SSTR2, with SSTR5 typically the second most expressed receptor [52, 53]. Some case series report association between SSTR5 expression and increased tumour aggressiveness [54]. Short and long‐acting forms of Octreotide and Lanreotide, which show highest affinity for SSTR2, have been shown not only to improve glycaemic control in patients with insulinoma [32, 55, 56], but also have been associated with disease stabilisation and regression [57, 58]. An in vitro study of Octreotide on rat insulinoma cells proposed inhibition of cell proliferation via the Akt‐mTOR pathway [59]. The glycaemic effects of short acting octreotide can occur as quickly as 6−8 h after injection, but variable efficacy has been reported, possibly due to differences in SSTR expression [60]. Tests such as SSTR imaging to demonstrate high density of SSTRs can be employed to identify patients who are more likely to have therapeutic benefit from treatment, but their reliability requires further evaluation, as there are several case reports of non‐response to somatostatin analogues despite high density of SSTR on Octreoscan [32, 56, 61]. Sustained reduction in hypoglycaemic episodes has been reported with a median treatment duration of 67 months in Vezzosi et al. [32, 33].

Pasireotide, a second‐generation somatostatin analogue with higher affinity for SSTR5 [35], has also been used in patients with insulinoma, showing sustained improvement in hypoglycaemia in some patients when other somatostatin analogues had been ineffective [36, 62, 63]. The effects of Pasireotide on tumour progression was evaluated by Cives et al. in patients with metastatic neuroendocrine tumours showing a median progression free survival of 11 months [37].

Side effects of somatostatin analogues include gastrointestinal disturbance, steatorrhoea, cholelithiasis and biliary disorders, and hyperglycaemia [32]. Paradoxical hypoglycaemia, possibly due to concurrent inhibition of glucagon release, has been reported on initiation and dose titration [64, 65] and therefore close monitoring of blood sugars and occasionally inpatient monitoring may be required during initiation of somatostatin analogues.

##### 2.3.2.3 Everolimus

Everolimus binds to the cyclophilin FKBP‐12 to form a complex which inhibits the mTOR signalling cascade. The mTOR cascade mediates diverse cellular effects via two distinct complexes mTORC1 and mTORC2 which have opposing actions for insulin secretion [66]. Activation of the mTOR signalling pathway, mediated through insulin‐like growth factor 1 (IGF‐1), has been implicated in the proliferation of neuroendocrine tumour cells and everolimus has been associated with improved progression free survival [41]. Furthermore, everolimus has been shown to suppress insulin secretion via inhibition of the mTORC1/S6K signalling pathway causing a reduction in hypoglycaemia in patients with advanced insulinomas [38, 39, 40, 67]. These therapeutic effects may function independently of one another, with everolimus showing continued reduction in hypoglycaemic events despite evidence of tumour progression [68]. Improvement in hypoglycaemia has been reported for patients who have failed multiple other therapeutic options, with everolimus started after a median of four other therapeutic avenues in a study by Bernard et al. [40]. The dose of everolimus is typically 5−10 mg daily, with improvement in hypoglycaemia seen within 3−14 days and sustained for several months [38, 39, 40]. Concurrent use with octreotide [39] and chemotherapy [69] has been reported. Side effects include rash, diarrhoea, fatigue, hyperglycaemia and increased susceptibility to infections [41].

##### 2.3.2.4 Glucocorticoids

Glucocorticoids inhibit insulin release and increase peripheral insulin resistance and have been used in the management of refractory hypoglycaemia at a low dose (prednisolone 2.5 mg daily, dexamethasone 0.5−4 mg) where other agents have been ineffective [61, 65]. They may also increase appetite and reduce nausea, though this should be carefully balanced with the side effects of long‐term steroid use including hypothalamic pituitary adrenal axis suppression.

#### 2.3.3 PRRT

Somatostatin receptor positivity is frequently preserved in metastatic insulinomas, allowing targeted treatment with somatostatin receptor‐based PRRT [70]. 177Lu‐DOTATATE (Lutathera), comprising the 8 amino acid peptide somatostatin receptor agonist DOTA‐TATE and 177‐Lutetium, was first approved by regulators in 2018 for the treatment of unresectable or metastatic well differentiated (G1 or G2) somatostatin receptor positive neuroendocrine tumours following the NETTER‐1 trial [71]. The NETTER‐1 trial has led to the recommendation of PRRT as a first line management approach for these patients alongside somatostatin analogue therapy, after primary analysis demonstrated prolongation of the median progression free survival by 14.3 months and significantly improved objective response rate compared with the control group receiving somatostatin analogue therapy alone [72].

The typical regimen for PRRT with 177Lu‐DOTATATE is 4 cycles of 7.4 GBq, given as an IV infusion at 8‐week intervals (total 29.6 GBq), though re‐treatment with additional cycles has been reported [73].

In addition to improvements in progression‐free survival for patients with insulinoma, PRRT has been reported to improve hypoglycaemia in 67−93% of patients with metastatic insulinomas [42, 43, 74, 75]. The time to achieve euglycaemia has not been fully evaluated, but cases in the literature suggest hypoglycaemia typically improves within 1−2 weeks of the first treatment cycle and can be sustained for up to 43 months [43, 44, 45, 76, 77, 78]. In a retrospective analysis by Friebe et al. 21 of 26 patients saw improvement in hypoglycaemia, with 17 (81%) experiencing improvement in hypoglycaemia after 1 cycle of PRRT and the remaining 4 patients seeing improvement after 2 cycles [42].

Patients undergoing PRRT commonly experience nausea and vomiting (59% and 47% respectively), with serious adverse effects including transient myelotoxicity (< 10%) [71]. The risk of myelotoxicity with PRRT may justify a dose reduction in concomitant cytoreductive therapies if used. Transient worsening of hypoglycaemia attributed to tumour lysis was also reported in 5 (19%) patients in a study by Friebe et al. [42], necessitating consideration for a period of 24−48 h inpatient observation post treatment in high‐risk individuals including those with large volume metastases or a history of difficult to control hypoglycaemia.

#### 2.3.4 Other Interventions

Treatments aimed at reducing the overall tumour burden, such as cytoreductive chemotherapy agents and locally targeted ablative treatments, may theoretically reduce overall insulin secretion and improve hypoglycaemia.

EUS guided radiofrequency or ethanol ablation of the primary insulinoma may be considered for patients in whom pancreatic surgery is inappropriate due to co‐morbidity, frailty or tumour characteristics. In a metanalysis by Xiao et al. endoscopic ablation had a shorter length of hospital stay but a higher rate of symptom recurrence when compared with minimally invasive surgery, but patients undergoing EUS‐guided ablation were older (64.06 vs. 44.98 years) [79].

Trans‐arterial chemo embolization (TACE) for hepatic metastases has been reported, though usually as part of a wider treatment regime including systemic therapy [69, 74]. Chemotherapeutic regimes including streptozocin, 5‐fluorouracil, capecitabine and temozolomide (CAPTEM) and cisplatin with etoposide have been used in the management of metastatic neuroendocrine tumour alongside everolimus and somatostatin analogues, though evidence for their independent effect on hypoglycaemia is lacking [36, 69, 77].

#### 2.3.5 End of Life Care

Evidence for best practice for patients with metastatic insulinoma at the end of life is lacking. Hypoglycaemia is a common cause of death for patients with metastatic insulinoma, whose disease has progressed despite multiple lines of therapy. Declining oral intake, anxiety around symptoms of hypoglycaemia, complex emotions regarding the cessation of CGM and difficulty in managing other symptoms such as pain should be navigated together with palliative care clinicians [80]. Individualised shared decision making is key and continuing medications such as somatostatin receptor antagonists (SSAs), glucocorticoids and diazoxide with the aim of alleviating symptoms may be helpful [81]. It may also be helpful to consider continuing enteral or PN if this in place, until a patient has reached their chosen destination (e.g., home or a hospice).

## 3 Discussion

**Figure 2:**
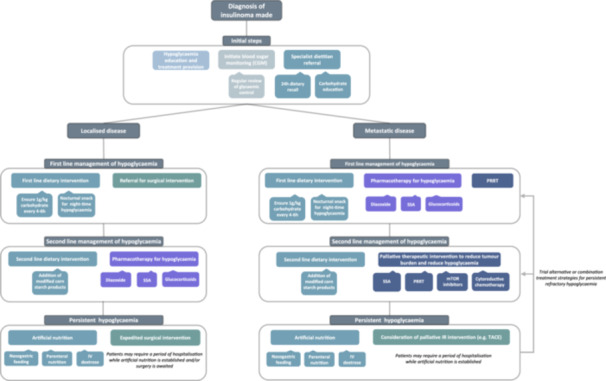
Proposed algorithm for the step‐wise management of hypoglycaemia in patients with insulinoma. IR, interventional radiology; IV, intravenous; PRRT, peptide receptor radionuclide therapy; SSA, somatostatin analogue; TACE, transarterial chemo‐embolisation.

Preventing life‐threatening hypoglycaemia must be the focus of care for patients with insulinoma, either while awaiting curative resection or during palliative treatment. We propose an algorithm for the management of these patients (Figure 2), highlighting the importance of multidisciplinary team working between medical, surgical and dietetic specialties.

Early education on hypoglycaemia identification and treatment for the patient and their family is critical. We advocate the use of continuous glucose monitoring in patients with insulinoma to reduce the incidence of severe hypoglycaemic episodes and enable dietary and medical intervention to be tailored for the individual and their lifestyle.

Patients with insulinoma should have specialist dietitian assessment and follow up comprising regular assessment of glycaemic control, nutritional education and intervention. Close communication between the dietitian and medical teams is essential. The first line strategy to reduce hypoglycaemia is to ensure adequate low‐GI carbohydrate intake (~1 g/Kg every 4−6 h) with the addition of nocturnal snacks if night‐time hypoglycaemia is problematic. The use of UCCS and modified cornstarch products mixed in a macronutrient rich vehicle substrate such as yoghurt can be considered.

In parallel, consideration should be given to surgical intervention or tumouristatic medical therapies which offer a reduction in tumour bulk ‐ not only to offer prognostic benefit, but to alleviate hypoglycaemia in the longer term for patients with metastatic disease. If medical therapy is indicated, somatostatin analogues should be considered first‐line due to their dual hyperglycaemic and tumouristatic effects. If hypoglycaemia persists, diazoxide and/or glucocorticoids can be added to the treatment regime. PRRT is an effective treatment offering improvement in progression free survival and long‐term improvement in hypoglycaemia and should be considered as a first line treatment approach in addition to somatostatin analogues in patients with metastatic or inoperable disease. Frequent review for emerging side effects and hyperglycaemia is necessary.

Episodes of severe hypoglycaemia may persist despite optimisation of carbohydrate intake and medical therapies. These patients may require a period of hospitalisation and IV dextrose admission while AN is established and/or definitive intervention is awaited. Refractory hypoglycaemia can be treated with a combination of oral carbohydrate optimisation, enteral and parenteral feeding in addition to a combination of medical therapies (SSA ± glucocorticoid ± diazoxide) as tolerated.

In summary, the maintenance of euglycaemia in patients with insulinoma can be challenging but is achievable with individualised specialist multidisciplinary care.

## Conflicts of Interest

The authors declare no conflicts of interest.

## References

[1] R. M. Wilder , F. N. Allan , M. H. Power , and H. E. Robertson , “Carcinoma of the Islands of the Pancreas: Hyperinsulinism and Hypoglycemia,” Journal of the American Medical Association 89, no. 5 (1927): 348–355, 10.1001/JAMA.1927.02690050014007.

[2] G. Howland , “Dysinsulinism: Convulsions and Coma Due to Islet Cell Tumor of the Pancreas, With Operation and Cure,” Journal of the American Medical Association 93, no. 9 (1929): 674–679, 10.1001/JAMA.1929.02710090014006.

[3] F. J. Service , M. M. McMAHON , P. C. O'brien , and D. J. Ballard , “Functioning Insulinoma—Incidence, Recurrence, and Long‐Term Survival of Patients: A 60‐year Study,” Mayo Clinic Proceedings 66, no. 7 (1991): 711–719, 10.1016/S0025-6196(12)62083-7. 1677058

[4] E. Peltola , P. Hannula , H. Huhtala , et al., “Characteristics and Outcomes of 79 Patients With an Insulinoma: A Nationwide Retrospective Study in Finland,” International Journal of Endocrinology 2018 (2018): 1–10, 10.1155/2018/2059481. PMC6218736 30425741

[5] A. Veltroni , E. Cosaro , F. Spada , et al., “Clinico‐Pathological Features, Treatments and Survival of Malignant Insulinomas: A Multicenter Study,” European Journal of Endocrinology 182, no. 4 (2020): 439–446, 10.1530/EJE-19-0989. 32061159

[6] J. Crona , O. Norlén , P. Antonodimitrakis , S. Welin , P. Stålberg , and B. Eriksson , “Multiple and Secondary Hormone Secretion in Patients With Metastatic Pancreatic Neuroendocrine Tumours,” The Journal of Clinical Endocrinology & Metabolism 101, no. 2 (2016): 445–452, 10.1210/JC.2015-2436. 26672633

[7] A. Munir , P. Choudhary , B. Harrison , S. Heller , and J. Newell‐Price , “Continuous Glucose Monitoring in Patients With Insulinoma,” Clinical Endocrinology 68, no. 6 (2008): 912–918, 10.1111/J.1365-2265.2007.03161.X. 18088393

[8] K. A. Placzkowski , A. Vella , G. B. Thompson , et al., “Secular Trends in the Presentation and Management of Functioning Insulinoma at the Mayo Clinic, 1987–2007,” Journal of Clinical Endocrinology & Metabolism 94, no. 4 (2009): 1069–1073, 10.1210/JC.2008-2031. 19141587

[9] D. Vezzosi , E. Guillaume , A. Bennet , C. Mouly , H. Hanaire , and P. Caron , “Medical Therapy in Patients With Endogenous Hypoglycaemia: Is Euglycaemia Achievable,” Clinical Endocrinology 90, no. 6 (2019): 798–804, 10.1111/CEN.13961. 30817011

[10] C. A. J. van Beers , J. H. DeVries , S. J. Kleijer , et al., “Continuous Glucose Monitoring for Patients With Type 1 Diabetes and Impaired Awareness of Hypoglycaemia (In Control): A Randomised, Open‐Label, Crossover Trial,” Lancet Diabetes & Endocrinology 4, no. 11 (2016): 893–902, 10.1016/S2213-8587(16)30193-0. 27641781

[11] B. Karges , S. R. Tittel , A. Bey , et al., “Continuous Glucose Monitoring Versus Blood Glucose Monitoring for Risk of Severe Hypoglycaemia and Diabetic Ketoacidosis in Children, Adolescents, and Young Adults With Type 1 Diabetes: A Population‐Based Study,” Lancet Diabetes & Endocrinology 11, no. 5 (2023): 314–323, 10.1016/S2213-8587(23)00061-X. 37004710

[12] Y. K. Lin , S. J. Fisher , and R. Pop‐Busui , “Hypoglycemia Unawareness and Autonomic Dysfunction in Diabetes: Lessons Learned and Roles of Diabetes Technologies,” Journal of Diabetes Investigation 11, no. 6 (2020): 1388–1402, 10.1111/JDI.13290. 32403204 PMC7610104

[13] R. Nakajima , H. Idesawa , D. Sato , et al., “Continuous Glucose Monitoring in a Patient With Insulinoma Presenting With Unawareness of Postprandial Hypoglycemia,” Endocrinology, Diabetes & Metabolism Case Reports 2023, no. 3 (2023), 10.1530/EDM-23-0056. PMC10563612 37767703

[14] T. M. S. Wolever , “Carbohydrate and the Regulation of Blood Glucose and Metabolism,” Nutrition Reviews 61, no. suppl_5 (2003): S40–S48, 10.1301/NR.2003.MAY.S40-S48. 12828191

[15] F. Brouns , I. Bjorck , K. N. Frayn , et al., “Glycaemic Index Methodology,” Nutrition Research Reviews 18, no. 1 (2005): 145–171, 10.1079/NRR2005100. 19079901

[16] F. J. White , “Inherited Metabolic Disorders,” In Clinical Paediatric Dietetics. (July, 2020), 502–512, 10.1002/9781119467205.CH27.

[17] M. Gallo , G. Muscogiuri , G. Pizza , et al., “The Management of Neuroendocrine Tumours: A Nutritional Viewpoint,” Critical Reviews in Food Science and Nutrition 59, no. 7 (2019): 1046–1057, 10.1080/10408398.2017.1390729. 29020456

[18] K. M. Ross , L. M. Brown , M. M. Corrado , et al., “Safety and Efficacy of Chronic Extended Release Cornstarch Therapy for Glycogen Storage Disease Type I,” JIMD Reports 26 (2016): 85–90, 10.1007/8904_2015_488. 26303612 PMC4864714

[19] W. Da , J. Rj , B. Ea , et al., “Short and Long‐Term Acceptability and Efficacy of Extended‐Release Cornstarch in the Hepatic Glycogen Storage Diseases: Results From the Glyde Study,” Orphanet Journal of Rare Diseases 19, no. 1 (2024): 1–13, 10.1186/S13023-024-03274-Y/TABLES/6. 38982397 PMC11232220

[20] R. R. Li , W. Chen , X. H. Xiao , M. Yu , F. Ping , and L. Duan , “Efficacy of Raw Corn Starch in Insulinoma‐Related Hypoglycemia: A Promising Supportive Therapy,” Chinese Medical Sciences Journal 39, no. 2 (2024): 102–110, 10.24920/004329. 38755752

[21] F. Fernández‐Bañares , “Carbohydrate Maldigestion and Intolerance,” Nutrients 14, no. 9 (2022): 1923, 10.3390/NU14091923. 35565890 PMC9099680

[22] V. Marks and E. Samols , “Glucagon Test for Insulinoma: a Chemical Study in 25 Cases,” Journal of Clinical Pathology 21, no. 3 (1968): 346–352, 10.1136/JCP.21.3.346. 4301688 PMC473794

[23] E. Baudin , P. Caron , C. Lombard‐Bohas , et al., “Malignant Insulinoma: Recommendations for Characterisation and Treatment,” Annales d'endocrinologie 74, no. 5–6 (2013): 523–533, 10.1016/J.ANDO.2013.07.001. 23993836

[24] J. Hofland , J. C. Refardt , R. A. Feelders , E. Christ , and W. W. de Herder , “Approach to the Patient: Insulinoma,” Journal of Clinical Endocrinology & Metabolism 109, no. 4 (2024): 1109–1118, 10.1210/CLINEM/DGAD641. 37925662 PMC10940262

[25] A. Matej , H. Bujwid , and J. Wroński , “Glycemic Control in Patients With Insulinoma,” Hormones 15, no. 4 (2017): 489–499, 10.14310/HORM.2002.1706. 28222404

[26] Z. Hofman , J. De Van Drunen , and H. Kuipers , “The Glycemic Index of Standard and Diabetes‐Specific Enteral Formulas,” Asia Pacific Journal of Clinical Nutrition 15, no. 3 (2006): 412–417. 16837435

[27] E. Niv , Z. Fireman , and N. Vaisman , “Post‐Pyloric Feeding,” World Journal of Gastroenterology 15, no. 11 (2009): 1281, 10.3748/WJG.15.1281. 19294757 PMC2658837

[28] T. Wu , S. S. Thazhath , C. S. Marathe , et al., “Comparative Effect of Intraduodenal and Intrajejunal Glucose Infusion on the Gut–Incretin Axis Response in Healthy Males,” Nutrition & Diabetes 5, no. 5 (2015): 156, 10.1038/nutd.2015.6. PMC4450461 25985092

[29] S. C. Bischoff , P. Austin , K. Boeykens , et al. ESPEN Guideline ESPEN Guideline on Home Enteral Nutrition. 2019, 10.1016/j.clnu.2019.04.022. 31255350

[30] Y. Niitsu , I. Minami , H. Izumiyama , et al., “Clinical Outcomes of 20 Japanese Patients With Insulinoma Treated With Diazoxide,” Endocrine Journal 66, no. 2 (2019): 149–155, 10.1507/ENDOCRJ.EJ18-0353. 30504655

[31] A. M. Warren , D. J. Topliss , and P. S. Hamblin , “Successful Medical Management of Insulinoma With Diazoxide for 27 Years,” Endocrinology, Diabetes & Metabolism Case Reports 2020, no. 1 (2020): 1–5, 10.1530/EDM-20-0132. PMC7576657 33434168

[32] D. Vezzosi , A. Bennet , F. Courbon , and P. Caron , “Short‐ and Long‐Term Somatostatin Analogue Treatment in Patients With Hypoglycaemia Related to Endogenous Hyperinsulinism,” Clinical Endocrinology 68, no. 6 (2008): 904–911, 10.1111/J.1365-2265.2007.03136.X. 18031316

[33] T. Katabami , H. Kato , N. Shirai , S. Naito , and N. Saito , “Successful Long‐Term Treatment With Once‐Daily Injection of Low‐Dose Octreotide in an Aged Patient With Insulinoma,” Endocrine Journal 52, no. 5 (2005): 629–634, 10.1507/ENDOCRJ.52.629. 16284444

[34] L. Verschoor , P. Uitterlinden , S. W. J. Lamberts , and E. Del Pozo , “On the Use of a New Somatostatin Analogue in the Treatment of Hypoglycaemia in Patients With Insulinoma,” Clin Endocrinol (Oxf) 25, no. 5 (1986): 555–560, 10.1111/J.1365-2265.1986.TB03609.X. 2887309

[35] H. A. Schmid , “Pasireotide (SOM230): Development, Mechanism of Action and Potential Applications,” Molecular and Cellular Endocrinology 286, no. 1–2 (2008): 69–74, 10.1016/J.MCE.2007.09.006. 17977644

[36] S. Oziel‐Taieb , J. Maniry‐Quellier , B. Chanez , F. Poizat , J. Ewald , and P. Niccoli , “Pasireotide for Refractory Hypoglycemia in Malignant Insulinoma‐ Case Report and Review of the Literature,” Frontiers in Endocrinology 13 (2022): 13, 10.3389/FENDO.2022.860614. PMC9063459 35518928

[37] M. Cives , P. L. Kunz , B. Morse , et al., “Phase Ii Clinical Trial of Pasireotide Long‐Acting Repeatable in Patients With Metastatic Neuroendocrine Tumors,” Endocrine‐related Cancer 22, no. 1 (2015): 1–9, 10.1530/ERC-14-0360. 25376618 PMC4643672

[38] H. B. Fiebrich , E. J. M. Siemerink , A. H. Brouwers , et al., “Everolimus Induces Rapid Plasma Glucose Normalization in Insulinoma Patients by Effects on Tumor As Well As Normal Tissues,” The Oncologist 16, no. 6 (2011): 783–787, 10.1634/THEONCOLOGIST.2010-0222. 21482586 PMC3228208

[39] M. Asayama , T. Yamada‐Murano , H. Hara , A. Ooki , M. Kurosumi , and K. Yamaguchi , “Everolimus Dramatically Improves Glycemic Control in Unresectable Metastatic Insulinoma: A Case Report,” Japanese Journal of Clinical Oncology 44, no. 2 (2014): 186–190, 10.1093/JJCO/HYT193. 24367043

[40] V. Bernard , C. Lombard‐Bohas , M. C. Taquet , et al., “Efficacy of Everolimus in Patients With Metastatic Insulinoma and Refractory Hypoglycemia,” European Journal of Endocrinology 168, no. 5 (2013): 665–674, 10.1530/EJE-12-1101. 23392213

[41] J. C. Yao , M. H. Shah , T. Ito , et al., “Everolimus for Advanced Pancreatic Neuroendocrine Tumors,” New England Journal of Medicine 364, no. 6 (2011): 514–523, 10.1056/NEJMOA1009290/SUPPL_FILE/NEJMOA1009290_DISCLOSURES.PDF. 21306238 PMC4208619

[42] L. Friebe , M. T. Freitag , M. Braun , et al., “Peptide Receptor Radionuclide Therapy Is Effective for Clinical Control of Symptomatic Metastatic Insulinoma: A Long‐Term Retrospective Analysis,” Journal of Nuclear Medicine 65, no. 2 (2024): 228–235, 10.2967/JNUMED.123.265894. 38164592 PMC13671384

[43] A. Bongiovanni , S. Nicolini , T. Ibrahim , et al., “177Lu‐DOTATATE Efficacy and Safety in Functioning Neuroendocrine Tumors: A Joint Analysis of Phase II Prospective Clinical Trials,” Cancers 14, no. 24 (2022): 6022, 10.3390/CANCERS14246022/S1. 36551507 PMC9776442

[44] P. Iglesias , A. Martínez , P. Gajate , T. Alonso , T. Navarro , and J. J. Díez , “Long‐Term Effect of 177LU‐DOTATATE on Severe and Refractory Hypoglycemia Associated With Malignant Insulinoma,” AACE Clinical Case Reports 5, no. 6 (2019): e330–e333, 10.4158/ACCR-2019-0086. 31967064 PMC6873842

[45] W. Makis , K. Mccann , and A. J. B. Mcewan , “Metastatic Insulinoma Pancreatic Neuroendocrine Tumor Treated With 177Lu‐DOTATATE Induction and Maintenance Peptide Receptor Radionuclide Therapy: A Suggested Protocol,” Clinical Nuclear Medicine 41, no. 1 (2016): 53–54, 10.1097/RLU.0000000000001023. 26562579

[46] S. L. Shyng , T. Ferrigni , and C. G. Nichols , “Regulation of KATP Channel Activity by Diazoxide and MgADP,” Journal of General Physiology 110 (1997): 643–654, http://www.jgp.org. 9382893 10.1085/jgp.110.6.643 PMC2229399

[47] G. V. Gill , O. Rauf , and I. A. MacFarlane , “Diazoxide Treatment for Insulinoma: A National Uk Survey,” Postgraduate Medical Journal 73, no. 864 (1997): 640–641, 10.1136/PGMJ.73.864.640. 9497974 PMC2431498

[48] Y. Komatsu , A. Nakamura , M. Takihata , et al., “Safety and Tolerability of Diazoxide in Japanese Patients With Hyperinsulinemic Hypoglycemia,” Endocrine Journal 63, no. 3 (2016): 311–314, 10.1507/ENDOCRJ.EJ15-0428. 26598136

[49] J. Adachi , M. Mimura , I. Minami , K. Kakihana , and T. Watanabe , “Thrombocytopenia Induced By Diazoxide in a Patient With an Insulinoma,” Internal Medicine 53, no. 7 (2014): 759–762, 10.2169/INTERNALMEDICINE.53.1094. 24694492

[50] A. J. Zillich , J. Garg , S. Basu , G. L. Bakris , and B. L. Carter , “Thiazide Diuretics, Potassium, and the Development of Diabetes: A Quantitative Review,” Hypertension 48, no. 2 (2006): 219–224, 10.1161/01.HYP.0000231552.10054.AA. 16801488

[51] M. Standard and H. Agent . PROGLYCEM (diazoxide) PROGLYCEM, accessed October 5, 2024, www.merck.ca.

[52] E. Peltola , T. Vesterinen , H. Leijon , et al., “Immunohistochemical Somatostatin Receptor Expression in Insulinomas,” APMIS 131, no. 4 (2023): 152–160, 10.1111/APM.13297. 36680557

[53] J. Bertherat , F. Tenenbaum , K. Perlemoine , et al., “Somatostatin Receptors 2 and 5 Are the Major Somatostatin Receptors in Insulinomas: An in Vivo and in Vitro Study,” Journal of Clinical Endocrinology & Metabolism 88, no. 11 (2003): 5353–5360, 10.1210/JC.2002-021895. 14602773

[54] S. de Sá , M. Corrêa‐Giannella , C. Machado , et al., “Somatostatin Receptor Subtype 5 (SSTR5) Mrna Expression Is Related to Histopathological Features of Cell Proliferation in Insulinomas,” Endocrine‐related Cancer 13, no. 1 (2006): 69–78, 10.1677/ERC.1.00962. 16601280

[55] F. E. Von Eyben , E. Grodum , H. J. Gjessing , C. Hagen , and H. B. Nielsen , “Metabolic Remission With Octreotide in Patients With Insulinoma,” Journal of Internal Medicine 235, no. 3 (1994): 245–248, 10.1111/J.1365-2796.1994.TB01067.X. 8120520

[56] D. Vezzosi , A. Bennet , P. Rochaix , et al., “Octreotide in Insulinoma Patients: Efficacy on Hypoglycemia, Relationships With Octreoscan Scintigraphy and Immunostaining With Anti‐sst2A and anti‐sst5 Antibodies,” European Journal of Endocrinology 152, no. 5 (2005): 757–767, 10.1530/EJE.1.01901. 15879362

[57] S. M. Longnecker , “Remission of Symptoms of Chemotherapy‐Refractory Metastatic Insulinoma Using Octreotide,” Drug Intelligence & Clinical Pharmacy 22, no. 2 (1988): 136–138, 10.1177/106002808802200207. 2894965

[58] S. Romeo , M. Milione , A. Gatti , et al., “Complete Clinical Remission and Disappearance of Liver Metastases After Treatment With Somatostatin Analogue in a 40‐Year‐Old Woman With a Malignant Insulinoma Positive for Somatostatin Receptors Type 2,” Hormone Research in Paediatrics 65, no. 3 (2006): 120–125, 10.1159/000091408. 16479142

[59] S. Grozinsky‐Glasberg , G. Franchi , M. Teng , et al., “Octreotide and the mTOR Inhibitor RAD001 (Everolimus) Block Proliferation and Interact With the Akt‐mTOR‐p70S6K Pathway in a Neuro‐Endocrine Tumour Cell Line,” Neuroendocrinology 87, no. 3 (2008): 168–181, 10.1159/000111501. 18025810

[60] No effect of the Long‐Acting Somatostatin Analogue Octreotide in Patients With Insulinoma—PubMed, accessed October 5, 2024, https://pubmed.ncbi.nlm.nih.gov/1922592/. 1922592

[61] J. Novotny , F. Janku , P. Mares , and L. Petruzelka , “Symptomatic Control of Hypoglycaemia With Prednisone in Refractory Metastatic Pancreatic Insulinoma,” Supportive Care in Cancer 13, no. 9 (2005): 760–762, 10.1007/S00520-005-0840-5/METRICS. 15959811

[62] A. Tirosh , S. Stemmer , E. Solomonov , et al., “Pasireotide for Malignant Insulinoma,” Hormones 15, no. 2 (2015): 271–276, 10.14310/HORM.2002.1639. 26732164

[63] M. Siddiqui , A. Vora , S. Ali , J. Abramowitz , and S. Mirfakhraee , “Pasireotide: A Novel Treatment for Tumor‐Induced Hypoglycemia Due to Insulinoma and Non‐Islet Cell Tumor Hypoglycemia,” Journal of the Endocrine Society 5, no. 1 (2021): 1–7, 10.1210/JENDSO/BVAA171. PMC7692539 33294765

[64] M. L. Healy , S. J. Dawson , R. M. L. Murray , J. Zalcberg , and M. Jefford , “Severe Hypoglycaemia After Long‐Acting Octreotide in a Patient With an Unrecognized Malignant Insulinoma,” Internal Medicine Journal 37, no. 6 (2007): 406–409, 10.1111/J.1445-5994.2007.01371.X. 17535385

[65] S. K. Abell , J. Teng , A. Dowling , M. S. Hofman , R. J. MacIsaac , and N. Sachithanandan , “Prolonged Life‐Threatening Hypoglycaemia Following Dose Escalation of Octreotide Lar in a Patient With Malignant Polysecreting Pancreatic Neuroendocrine Tumour,” Endocrinology, Diabetes & Metabolism Case Reports 2015, 14 no. 1 (2015): 14, 10.1530/EDM-14-0097. PMC4313612 25755880

[66] V. Panwar , A. Singh , M. Bhatt , et al., “Multifaceted Role of Mtor (Mammalian Target of Rapamycin) Signaling Pathway in Human Health and Disease,” Signal Transduction and Targeted Therapy 8, no. 1 (2023): 375, 10.1038/S41392-023-01608-Z. 37779156 PMC10543444

[67] J. Ferrer‐García , M. Tolosa‐Torréns , C. Hernando‐Meliá , L. Arribas‐Palomar , and C. Sánchez‐Juan , “Everolimu Resolving Hypoglycemia, Producing Hyperglycemia, and Necessitating Insulin Use in A Patient With Di Etes and Nonresectable Malignant Insulinoma,” Endocrine Practice 17, no. 2 (2011): e17–e20, 10.4158/EP10282.CR. 21247848

[68] L. Suzuki , T. Miyatsuka , M. Himuro , et al., “Everolimus Directly Suppresses Insulin Secretion Independently of Cell Growth Inhibition,” Journal of the Endocrine Society 2, no. 7 (2018): 589–596, 10.1210/JS.2017-00475. 29942923 PMC6007247

[69] M. Scharf , D. Mueller , U. Koenig , et al., “Management of a Metastasized High Grade Insulinoma (G3) With Refractory Hypoglycemia: Case Report and Review of the Literature,” Pancreatology 14, no. 6 (2014): 542–545, 10.1016/J.PAN.2014.07.011. 25459566

[70] D. Wild , E. Christ , M. E. Caplin , et al., “Glucagon‐Like Peptide‐1 Versus Somatostatin Receptor Targeting Reveals 2 Distinct Forms of Malignant Insulinomas,” Journal of Nuclear Medicine 52, no. 7 (2011): 1073–1078, 10.2967/JNUMED.110.085142. 21680696

[71] J. Strosberg , G. El‐Haddad , E. Wolin , et al., “Phase 3 Trial of 177 Lu‐Dotatate for Midgut Neuroendocrine Tumors,” New England Journal of Medicine 376, no. 2 (2017): 125–135, 10.1056/NEJMOA1607427/SUPPL_FILE/NEJMOA1607427_DISCLOSURES.PDF. 28076709 PMC5895095

[72] S. Singh , D. M. Halperin , S. Myrehaug , et al., “177Lu]Lu‐DOTA‐TATE in Newly Diagnosed Patients With Advanced Grade 2 and Grade 3, Well‐Differentiated Gastroenteropancreatic Neuroendocrine Tumors: Primary Analysis of the Phase 3 Randomized NETTER‐2 Study,” Journal of Clinical Oncology 42, no. 3_suppl (2024): LBA588‐LBA588, 10.1200/JCO.2024.42.3_SUPPL.LBA588. 38851203

[73] C. Unal , “Assessing the Clinical Impact of Lutetium‐177 DOTATATE Peptide Receptor Radionuclide Therapy (PRRT) on Metastatic Neuroendocrine Tumors: A Multicenter Real‐World Data From Türkiye,” Eurasian Journal of Medicine and Oncology 7, no. 3 (2023): 232–242, 10.14744/EJMO.2023.48337/PDF/.

[74] A. Veltroni , E. Cosaro , F. Spada , et al., “Clinico‐Pathological Features, Treatments and Survival of Malignant Insulinomas: A Multicenter Study,” European Journal of Endocrinology 182, no. 4 (2020): 439–446, 10.1530/EJE-19-0989. 32061159

[75] W. T. Zandee , T. Brabander , A. Blažević , et al., “Symptomatic and Radiological Response to 177Lu‐DOTATATE for the Treatment of Functioning Pancreatic Neuroendocrine Tumors,” Journal of Clinical Endocrinology & Metabolism 104, no. 4 (2019): 1336–1344, 10.1210/JC.2018-01991. 30566620

[76] E. Van Schaik , E. I. Van Vliet , R. A. Feelders , et al., “Improved Control of Severe Hypoglycemia in Patients With Malignant Insulinomas by Peptide Receptor Radionuclide Therapy,” Journal of Clinical Endocrinology & Metabolism 96, no. 11 (2011): 3381–3389, 10.1210/JC.2011-1563. 21917872

[77] T. Terashima , T. Yamashita , N. Takemura , et al., “A Case of Frequent Hypoglycemic Attacks Successfully Controlled With Capecitabine Plus Temozolomide and 177Lu‐DOTATATE Peptide Receptor Radionuclide Therapy in a Patient With Recurrent Pancreatic Insulinoma,” Clinical Journal of Gastroenterology 16, no. 5 (2023): 767–771, 10.1007/S12328-023-01824-8. 37405635

[78] F. Novruzov , L. Mehmetbeyli , J. A. Aliyev , B. Abbasov , and E. Mehdi , “Metastatic Insulinoma Controlled by Targeted Radionuclide Therapy With 177Lu‐DOTATATE in a Patient With Solitary Kidney and MEN‐1 Syndrome,” Clinical Nuclear Medicine 44, no. 6 (2019): e415–e417, 10.1097/RLU.0000000000002500. 30789400

[79] D. Xiao , L. Zhu , S. Xiong , et al., “Outcomes of Endoscopic Ultrasound‐Guided Ablation and Minimally Invasive Surgery in the Treatment of Pancreatic Insulinoma: A Systematic Review and Meta‐Analysis,” Front Endocrinol (Lausanne) 15 (2024): 1367068, 10.3389/FENDO.2024.1367068/BIBTEX. 38645424 PMC11026617

[80] L. Simkiss , F. Hakkak , and R. Raghavan , “Malignant Insulinoma Hypoglycaemia: Complex Palliative Management,” BMJ Supportive & Palliative Care 14 (2021): e1772–e1774, 10.1136/BMJSPCARE-2021-003188. 34162582

[81] J. Del Rivero , J. Mailman , M. W. Rabow , et al., “Practical Considerations When Providing Palliative Care to Patients With Neuroendocrine Tumors in the Context of Routine Disease Management or Hospice Care,” Endocrine‐Related Cancer 30, no. 7 (2023): e220226, 10.1530/ERC-22-0226. 37017232 PMC10326633

